# Real-world patient-reported outcome comparing antibody–drug conjugates and conventional chemotherapy in metastatic breast cancer

**DOI:** 10.1093/oncolo/oyag394

**Published:** 2026-10-05

**Authors:** Manuela Milani, Francesco Riccetelli, Carla Strina, Marzia Alberio, Valeria Cervoni, Nicoletta Ziglioli, Donatella Caffi, Francesca Ferrè, Massimo Di Maio, Gabriella Pravettoni, Daniele Generali

**Affiliations:** SC Oncologia 2 Multidisciplinare—Azienda Socio-Sanitaria di Cremona, Cremona, 26100, Italy; SC Oncologia 2 Multidisciplinare—Azienda Socio-Sanitaria di Cremona, Cremona, 26100, Italy; SC Oncologia 2 Multidisciplinare—Azienda Socio-Sanitaria di Cremona, Cremona, 26100, Italy; SC Oncologia 2 Multidisciplinare—Azienda Socio-Sanitaria di Cremona, Cremona, 26100, Italy; SC Oncologia 2 Multidisciplinare—Azienda Socio-Sanitaria di Cremona, Cremona, 26100, Italy; SC Oncologia 2 Multidisciplinare—Azienda Socio-Sanitaria di Cremona, Cremona, 26100, Italy; SC Oncologia 2 Multidisciplinare—Azienda Socio-Sanitaria di Cremona, Cremona, 26100, Italy; Dipartimento di Scienze Biomediche per la Salute, University of Milan, Milan, 20122, Italy; Department of Oncology, University of Turin, at A.O. Ordine Mauriziano Hospital, Turin, 10124, Italy; Department of Oncology and Hemato-Oncology, University of Milan, Milan, 20122, Italy; SC Oncologia 2 Multidisciplinare—Azienda Socio-Sanitaria di Cremona, Cremona, 26100, Italy

**Keywords:** breast neoplasms, immunoconjugates, patient-reported outcome measures, quality of life, caregiver burden, neoplasm metastasis

## Abstract

**Background:**

Antibody–drug conjugates (ADCs) have transformed second-line metastatic breast cancer (MBC) therapy, yet their multidimensional quality-of-life impact vs conventional intravenous chemotherapy in real-world practice, particularly on lifestyle and caregiver burden, remains poorly characterized.

**Materials and methods:**

This prospective cohort enrolled 122 consecutive patients with MBC starting second-line therapy: 51 received ADCs and 71 conventional intravenous chemotherapy, allocated by tumor biology and clinical factors. The DAMA (Digital Antibody drug conjugate Modulator in breAst cancer) framework collected electronic patient-reported outcome measures (ePROMs) on anthropometric, lifestyle, symptom, psychosocial, and caregiver-burden domains at baseline, 3 and 6 months, analyzed with linear mixed-effects models.

**Results:**

At baseline, measured lifestyle, symptom, psychosocial, and caregiver variables were broadly similar between cohorts, although treatment-related characteristics differed. At 3 months, ADC patients had greater mean weight loss (−3.1 vs −1.2 kg), a larger decline in weekly physical activity (−41 vs −22 min/wk), higher stress and caregiver burden, but lower pain scores (3.7 vs 5.3). By 6 months, several differences attenuated. In pooled descriptive analyses, weekly physical activity showed little association with pain (*r* = 0.03, *P* = .56), stress (*r* = −0.12, *P* = .03), anxiety (*r* = −0.02, *P* = .73), caregiver emotional wellbeing (*r* = 0.00, *P* = .97), or sleep duration (*r* = 0.04, *P* = .47).

**Conclusion:**

In this prospective real-world cohort, ADC and conventional chemotherapy cohorts showed different longitudinal patterns of patient- and caregiver-reported burden. Because treatment allocation was biology- and clinically driven, these findings are exploratory and should not be interpreted as causal treatment effects. Systematic ePROM collection within the DAMA framework was feasible and may support identification of supportive-care needs.

Implications for practice In this real-world cohort, several patient- and caregiver-reported outcomes differed between clinically distinct ADC and chemotherapy groups during the first 6 months. These exploratory associations should not be interpreted as causal effects of ADC treatment. The first 3 months may represent an important window for proactive multidisciplinary support, including nutritional counseling, exercise oncology, psycho-oncology, and caregiver-centered interventions. Routine integration of structured electronic patient-reported outcome measures, such as the DAMA framework, is feasible in community Breast Units and may help identify domains requiring individualized supportive care and shared decision-making.

## Introduction

Metastatic breast cancer (MBC) remains incurable in the vast majority of cases but is increasingly manageable, with progressive survival improvements attributable to therapeutic innovations.^1^^,^^2^ Antibody–drug conjugates (ADCs), combining monoclonal antibody specificity with cytotoxic payloads, have transformed treatment paradigms for HER2-positive and triple-negative breast cancer (TNBC).^3^^,^^4^ Trastuzumab emtansine (T-DM1), trastuzumab deruxtecan (T-DXd), and sacituzumab govitecan (SG) demonstrated significant progression-free survival (PFS) and overall survival (OS) advantages over established therapies in randomized controlled trials.^5^^,^^6^

However, clinical trial populations may underrepresent real-world patients’ heterogeneity in terms of functional status, comorbidities, and psychosocial needs.^7^^,^^8^ ADCs present unique toxicity profiles—including gastrointestinal symptoms, fatigue, myelosuppression, and also interstitial lung disease (ILD) for T-DXd, potentially impacting daily functioning, lifestyle behaviors, and caregiver dynamics.^3^^,^^9^^,^^10^ Standard clinical trial reporting emphasizes investigator-reported endpoints, such as tumor response, PFS, OS, and Common Terminology Criteria for Adverse Events (CTCAE) grade 3-4 toxicities, but may not fully reflect the impact of disease and treatment on patients’ daily lives.^11^^,^^12^ Patient-reported outcome measures (PROMs) provide complementary perspectives, capturing symptoms, functional status, and quality of life without clinician interpretation.^13^ Landmark studies demonstrate that systematic PROM collection with clinical integration improves symptom control, reduces healthcare utilization, and remarkably, enhances survival.^14–16^ Substantial discordance exists between clinician-assessed and patient-reported symptoms, particularly for subjective domains including fatigue, pain, and psychological distress.^13^^,^^17^^,^^18^

Despite ADC therapeutic advances, comprehensive real-world evaluations comparing their multidimensional impact vs conventional chemotherapy remain limited. Critical knowledge gaps persist regarding longitudinal changes in lifestyle factors (nutrition, physical activity, sleep), psychosocial wellbeing (pain, sexuality, anxiety, stress, social relationships), caregiver burden, and longitudinal patterns over time.^18^^,^^19^

To address these gaps, we implemented the DAMA (Digital Antibody drug conjugate Modulator in breAst cancer), a structured, comprehensive digital PROM system integrated into routine clinical workflows at “SS Oncologia 2 Multidisciplinare”- Breast Unit of ASST Cremona. This prospective real-world study described longitudinal patient- and caregiver-reported outcomes across clinically distinct ADC and conventional intravenous chemotherapy cohorts across multidimensional patient and caregiver outcomes during the first 6 months of second-line MBC treatment.

## Materials and methods

### Study design and setting

This prospective observational cohort study enrolled consecutive MBC patients initiating second-line systemic therapy at the Breast Unit of the ASST Cremona, Lombardy, Italy, between September 1, 2023 and September 30, 2025. The study received the ASST Cremona Ethics Committee approval (IRB nr. 34326) and adhered to Declaration of Helsinki principles. All participants provided written informed consent, including consent for caregiver participation. The patient population was selected by the following eligibility criteria:

- Age ≥18 years with histologically confirmed MBC;
- Initiating second-line systemic therapy (ADC or standard intravenous chemotherapy) Eastern Cooperative Oncology Group (ECOG) performance status ≤2;
- Life expectancy ≥6 months per treating oncologist assessment;
- Ability to complete Italian-language PROM assessments with interpreter support if needed;
- Identified primary caregiver willing to participate.

The treatment assignment was made by the multidisciplinary breast tumor board based on tumor biology, ECOG status, comorbidities, and toxicity history.

The ADC Cohort (*n* = 51) was composed by:

- Trastuzumab deruxtecan (*n* = 9): 5.4 mg/kg intravenously every 3 weeks for HER2-positive MBC following first-line HER2-directed therapy progression;
- T-DM1 (*n* = 19): 3.6 mg/kg intravenously every 3 weeks for HER2-positive MBC following first-line HER2-directed therapy progression;
- Sacituzumab govitecan (*n* = 23): 10 mg/kg intravenously on days 1 and 8 of 21-day cycles for TNBC following first-line chemotherapy progression.

The Standard Intravenous Chemotherapy Cohort (*n* = 71) was composed by:

- Single-agent taxanes (paclitaxel; *n* = 24, 33.8%);
- Eribulin mesylate (*n* = 18, 25.4%) (+Trastuzumab in HER2 + ve setting);
- Metronomic capecitabine/vinorelbine (*n* = 20, 28.1%);
- Carboplatin-based regimens (*n* = 9, 12.7%) (+Trastuzumab in HER2 + ve setting).

### The DAMA assessment framework

Comprehensive ePROMs assessments occurred at 3 timepoints: baseline (T0, within 1 week pre-treatment), 3 months (T1, ±2 weeks), and 6 months (T2, ±2 weeks). A trained breast-unit research nurse conducted standardized 30-40-minute face-to-face interviews, during which the patients learned about ePROMs and how to use the care@you electronic diary. The nurse followed structured protocols, scheduled to coincide with treatment visits to minimize patient burden.

#### Assessment domains

Anthropometric measures: Weight (kg), height (cm, baseline only), BMI (kg/m²), with clinically significant weight loss defined as ≥5% baseline weight.

##### Lifestyle factors

Physical activity: International Physical Activity Questionnaire short form, assessing type, frequency, duration, and intensity; classified as sedentary (<30 min/wk), insufficient (30-149 min/wk), or sufficient (≥150 min/wk per WHO guidelines).^20^

Nutrition: Quality and quantity assessment for protein, carbohydrates, and fiber; adequate fiber defined as ≥25 g daily for women.

Sleep: Duration (hours/night) and quality (good/fair/poor based on restfulness and nocturnal awakenings).

Substance use: Current tobacco (yes/no, cigarettes/day) and alcohol consumption (frequency, quantity).

##### Symptom burden

Pain: Numerical Rating Scale (NRS 0-10), with 0 = no pain, 1-3 = mild, 4-6 = moderate, 7-10 = severe; average pain over past week assessed.

##### Psychosocial wellbeing

Sexuality: Function and satisfaction categorized as no concerns, mild-moderate concerns, or severe concerns (informed by the EORTC QLQ-BR45^21^).

Social relationships: Quality and frequency rated as robust, adequate, limited, or isolated (informed by the EORTC QLQ-BR45^21^).

Stress: Perceived Stress Scale (PSS)^22^ adapted 1-20 range (1-5 = minimal, 6-10 = mild, 11-15 = moderate, 16-20 = severe).

Anxiety: Generalized Anxiety Disorder (GAD-7) total score (0-21); higher scores indicate greater anxiety.^23^ A separate normalized 0-10 representation was retained only as a derived variable and was not used for the primary inferential analysis.

##### Caregiver burden

Primary caregivers completed 5 study-specific adapted caregiver-burden domains informed by the Zarit Burden Interview^24^; these domains were not formally validated in this study and are reported descriptively.

Temporal assistance (1-20 scale): Time commitment for appointments, medication management, symptom monitoring, ADL assistance;
Quality of life (1-20 scale): Impact on caregiver life satisfaction;
Physical fatigue (0-16 scale): Somatic caregiving burden;
Social functioning (0-20 scale): Impact on social relationships and activities;
Emotional wellbeing (1-20 scale): Psychological state and emotional health.

### Data management and quality assurance

Data were collected digitally using the care@you electronic diary application by the patient to ensure consistency at all 3 assessment timepoints (T0, T1 at 3 months, and T2 at 6 months). Patients received pop-up alerts on their personal mobile phones. Responses were entered directly into the care@you/Sicuro platform and stored in the DICON blockchain-secured database. Research coordinators underwent standardized training with inter-rater reliability assessment. Monthly data quality reviews identified missing data patterns and implemented corrective actions.

### Statistical analysis

#### Sample size

This pragmatic study enrolled all eligible consecutive patients during the study period. No formal sample-size calculation was performed; the cohort size reflects the consecutive eligible population during the study period.

#### Longitudinal analysis

Repeated PROM observations were analyzed using linear mixed-effects models with a patient-level random intercept, categorical time (T0, T1, and T2), treatment group, and the time-by-treatment interaction, fitted by restricted maximum likelihood using all available observations. The treatment-by-time interaction was the prespecified inferential term for differences in longitudinal trajectories. Binary outcomes were analyzed with generalized linear mixed-effects models of the same structure where stable estimation was possible; sparse binary outcomes were reported descriptively. No silent imputation was performed. Because treatment assignment was determined by tumor biology and clinical factors and subtype was collinear with treatment, the models are interpreted as exploratory associations rather than causal treatment effects.

#### Multiplicity

For the 14 baseline between-group comparisons of lifestyle, psychosocial, and caregiver variables, two-sided *P* values were adjusted using the Benjamini–Hochberg false discovery rate procedure^25^ (*q* values), which are reported alongside the nominal *P* values. The longitudinal mixed-effects analyses and exploratory correlation analyses were interpreted using nominal two-sided *P* values; no global multiplicity correction was applied to the correlation analyses because they were exploratory.

#### Descriptive statistics

Baseline characteristics were compared using chi-square/Fisher’s exact tests (categorical variables), independent *t* tests (normally distributed continuous variables), or Mann–Whitney *U* tests (non-normally distributed continuous variables). Data are presented as mean (SD) or *n* (%).

#### Correlation analysis

Spearman rank correlation coefficients assessed associations between weekly moderate-intensity physical activity minutes and patient/caregiver outcomes (Pain NRS, stress, anxiety, caregiver emotional wellbeing, and sleep duration). Coefficients were estimated separately at T0 (*n* = 122), T1 (*n* = 118), and T2 (*n* = 108) to avoid pseudo-replication; pooled estimates (*n* = 348 observations) were retained as a descriptive complement. Per-timepoint coefficients are reported in Table S1. Two-tailed asymptotic *P* values are reported without global multiplicity correction because these analyses were exploratory.

#### Statistical significance

Statistical significance was set at *P* < .05 (2-tailed). Analyses used Stata 17.0 (StataCorp, College Station, TX).

## Results

### Patient flow and characteristics

Of 150 consecutive MBC patients screened, 122 (81.3%) completed baseline DAMA assessment and comprised the analysis cohort (Figure 1). The ePROMs were obtained using the care@you electronic diary application and stored in the DICON protected database. Twenty-eight patients (18.7%) did not complete the ePROMs due to refusal while accepting treatment (*n* = 12), unwillingness to continue (*n* = 11), rapid clinical deterioration (*n* = 3), or cognitive impairment (*n* = 2). Non-completers had higher ECOG 2 status (35.7% vs 15.6%, *P* = .02) and lower education (64.3% vs 44.3% secondary or less, *P* = .048), suggesting that the analysis cohort represents somewhat better baseline-status patients. Follow-up completion was 96.7% (*n* = 118) at 3 months and 88.5% (*n* = 108) at 6 months, with no significant difference between groups (ADC: 94.1% at T1, 86.3% at T2; chemotherapy: 98.6% at T1, 90.1% at T2; *P* = .24). At T2, 7 ADC patients and 7 chemotherapy patients had missing PROM assessments. In the ADC group, 3 were attributed to disease progression and 4 to loss to follow-up; in the chemotherapy group, 4 were attributed to disease progression and 3 to loss to follow-up. Absolute numbers of assessments completed per cohort were T1: ADC *n* = 48 and chemotherapy *n* = 70; T2: ADC *n* = 44 and chemotherapy *n* = 64. Treatment discontinuations were recorded separately from missing PROM assessments; documented missing PROM assessments and intercurrent events are detailed in Table S2.

**Figure 1.**
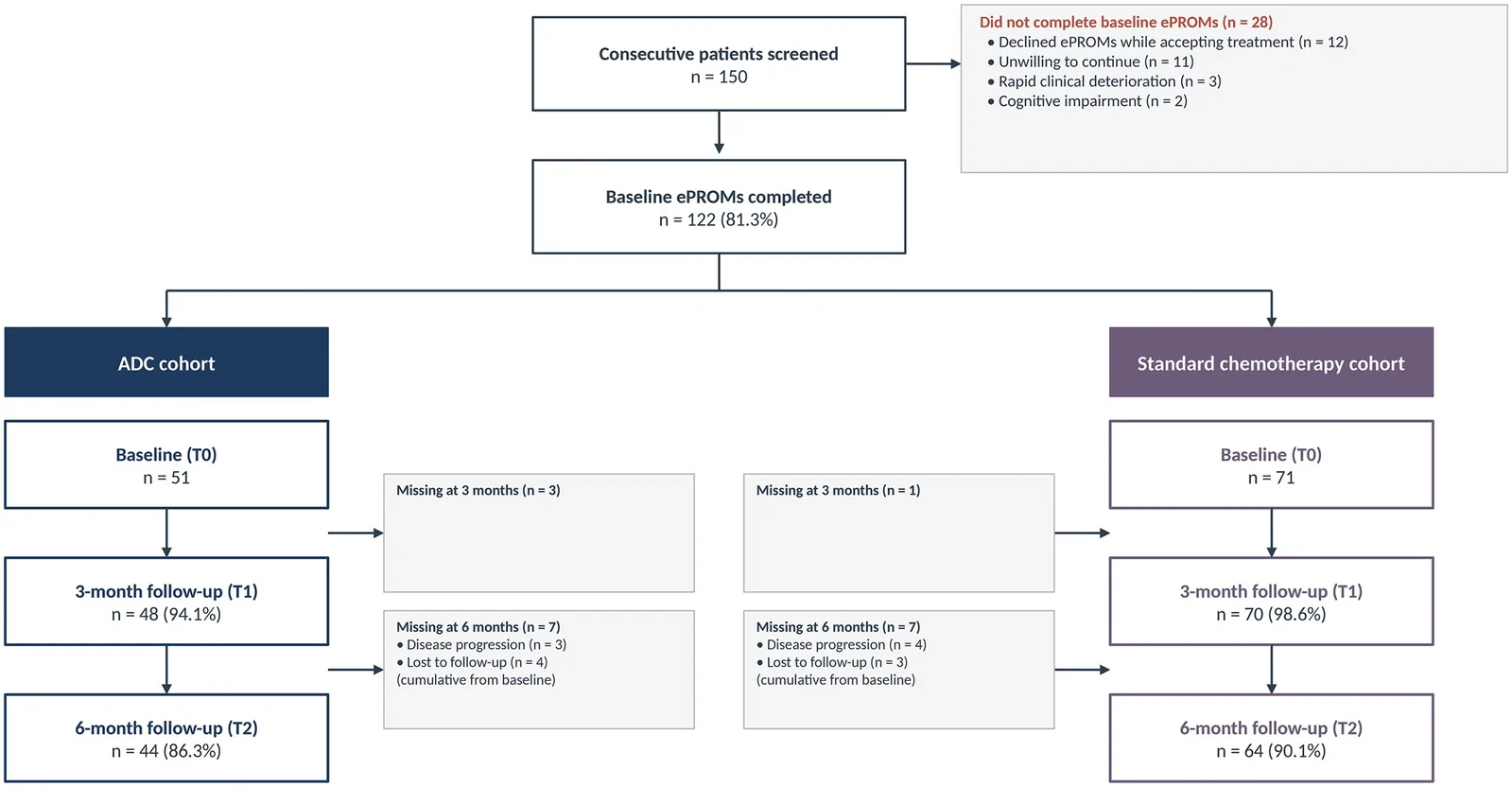
DAMA CONSORT patient flow diagram. ADC, antibody–drug conjugate; ePROMs, electronic patient-reported outcome measures; T0, baseline; T1, 3-month assessment; T2, 6-month assessment. Missing assessments at 6 months are cumulative from baseline.

Baseline characteristics are summarized in Table 1. Mean age was 58.8 (8.8) years in the ADC cohort and 59.6 (9.4) years in the chemotherapy cohort. The ADC cohort comprised HER2-positive (54.9%) and TNBC (45.1%) disease, whereas the chemotherapy cohort comprised HER2-positive (19.7%), HR+/HER2-negative (45.1%), and TNBC (35.2%). ECOG distributions were 43.1%, 45.1%, and 11.8% for scores 0, 1, and 2 in the ADC cohort and 42.3%, 45.1%, and 12.7% in the chemotherapy cohort. Variables not represented in the analytical dataset were not included in the revised table.

**Table 1.** Baseline patient and disease characteristics.

Characteristics	ADC therapy (*n* = 51)	Standard IV chemo (*n* = 71)	*P*-value
**Demographics**			
** Age, mean (SD), years**	58.8 (8.8)	59.6 (9.4)	.63
** Age ≥65 years, *n* (%)**	12 (23.5)	22 (31.0)	.42
**Breast cancer subtype**			<.001
** HR+/HER2-**	0 (0.0)	32 (45.1)	
** HER2+**	28 (54.9)	14 (19.7)	
** Triple-negative**	23 (45.1)	25 (35.2)	
**ECOG performance status**			.99
** 0**	22 (43.1)	30 (42.3)	
** 1**	23 (45.1)	32 (45.1)	
** 2**	6 (11.8)	9 (12.7)	

Abbreviations: ADC, antibody–drug conjugate; Chemo, chemotherapy; ECOG, Eastern Cooperative Oncology Group; HER2, human epidermal growth factor receptor 2; HR, hormone receptor; IV, intravenous; SD, standard deviation.

### Baseline lifestyle, psychosocial, and caregiver burden

Baseline assessments revealed substantial burden across both cohorts without major between-group differences in the available PROM variables (Table 2). At baseline, 43.1% of ADC-treated and 23.9% of chemotherapy-treated patients met ≥150 min/wk of activity, while 5.9% and 5.6%, respectively, were sedentary (<30 min/wk).

**Table 2.** Baseline lifestyle, psychosocial factors, and caregiver burden.

Domain	ADC (*n* = 51)	Standard Chemo (*n* = 71)	*P*-value	*q* value
**Anthropometrics**				
** Weight, mean (SD), kg**	67.8 (13.9)	70.4 (15.2)	.32	0.66
** BMI, mean (SD), kg/m²**	26.2 (5.7)	26.5 (5.9)	.74	0.74
**Physical activity**				
** Sedentary (<30 min/wk), *n* (%)**	3 (5.9)	4 (5.6)	.07	0.66
** Insufficient (30-149 min/wk), *n* (%)**	26 (51.0)	50 (70.4)		
** Sufficient (≥150 min/wk), *n* (%)**	22 (43.1)	17 (23.9)		
**Sleep**				
** Duration, mean (SD), hours**	6.6 (0.8)	6.5 (0.9)	.52	0.66
**Psychosocial**				
** Pain NRS [0–10], mean (SD)**	3.4 (2.5)	3.9 (2.7)	.29	0.66
** Stress [1–20], mean (SD)**	12.6 (4.3)	12.1 (4.5)	.53	0.66
** Anxiety [0–21], mean (SD)**	5.9 (2.2)	5.6 (2.3)	.46	0.66
** Moderate-severe anxiety (≥4), *n* (%)**	46 (90.2)	59 (83.1)	.30	0.66
** Sexual function concerns, *n* (%)**	22 (43.1)	40 (56.3)	.20	0.66
**Caregiver burden**				
** Temporal assistance [1–20], mean (SD)**	8.6 (3.7)	8.2 (3.9)	.58	0.66
** Quality of life [1–20], mean (SD)**	13.1 (3.9)	13.6 (3.7)	.48	0.66
** Physical fatigue [0–16], mean (SD)**	7.4 (3.5)	7.0 (3.7)	.54	0.66
** Social functioning [0–20], mean (SD)**	10.5 (3.2)	10.8 (3.2)	.61	0.66
** Emotional wellbeing [1–20], mean (SD)**	10.6 (3.2)	10.9 (3.2)	.61	0.66

Abbreviations: ADC, antibody–drug conjugate; BMI, body mass index; GAD-7, Generalized Anxiety Disorder-7; NRS, Numerical Rating Scale; SD, standard deviation. *q* values are Benjamini–Hochberg adjusted *P* values for the family of 14 baseline between-group comparisons (*m* = 14).

Baseline sleep measures were also broadly similar between cohorts. Mean sleep duration was 6.6 hours (SD 0.8) in the ADC cohort and 6.5 hours (SD 0.9) in the chemotherapy cohort (*P* = .52; *q* = .66), both below the commonly recommended 7-9 hr/d.

Baseline pain burden was moderate (mean NRS 3.4 ADC vs 3.9 chemotherapy, *P* = .29), with approximately half (50.8%) reporting moderate-to-severe pain (NRS ≥4). Sexual function concerns were reported by 43.1% of ADC-treated and 56.3% of chemotherapy-treated patients. Stress levels indicated moderate burden (mean 12.6 vs 12.1 on the 1-20 scale), and 90.2% of ADC and 83.1% of chemotherapy patients had GAD-7 total scores ≥4 on the 0-21 scale.

Baseline caregiver burden was moderate without significant between-group differences across temporal assistance (8.6 vs 8.2), quality of life (13.1 vs 13.6), physical fatigue (7.4 vs 7.0), social functioning (10.5 vs 10.8), and emotional wellbeing (10.6 vs 10.9) domains.

### Three-month outcomes: acute treatment impact

At 3 months, observed between-cohort differences were present across several domains (Table 3). These timepoint summaries are descriptive; formal inference on longitudinal differences is based on the treatment-by-time interaction from the mixed-effects models.

**Table 3.** Three-month outcomes: observed descriptive summaries.

Outcome	ADC (*n* = 48)	Standard Chemo (*n* = 70)
**Anthropometric changes**		
** Weight change from baseline, mean (SD), kg**	−3.1 (1.4)	−1.2 (1.1)
** Clinically significant weight loss (≥5%), *n* (%)**	21 (43.8)	4 (5.7)
**Physical activity**		
** Meeting guidelines (≥150 min/wk), *n* (%)**	7 (14.6)^a^	17 (24.3)^a^
** Change in weekly activity, mean (SD), min**	−41 (82)	−22 (101)
**Sleep**		
** Poor quality, *n* (%)**	20 (41.7)	20 (28.6)
**Symptom burden**		
** Pain NRS, mean (SD)**	3.7 (1.9)	5.3 (2.1)
** Change from baseline, mean (SD)**	+0.2 (3.1)	+1.4 (3.5)
**Psychosocial**		
** Stress score, mean (SD)**	14.1 (4.6)	11.5 (4.0)
** Change from baseline, mean (SD)**	+1.6 (7.4)	−0.6 (6.5)
** Anxiety score, mean (SD)**	9.1 (4.3)	7.2 (4.1)
** Robust/adequate social relationships, *n* (%)**	39 (81.3)	62 (88.6)
**Caregiver burden**		
** Temporal assistance, mean (SD)**	12.0 (4.5)	9.5 (3.8)
** Change from baseline, mean (SD)**	+3.4 (6.9)	+1.3 (5.5)
** Physical fatigue, mean (SD)**	10.0 (3.9)	7.8 (3.6)
** Change from baseline, mean (SD)**	+2.4 (5.2)	+0.8 (5.0)
** Quality of life, mean (SD)**	10.7 (3.7)	12.7 (3.5)
** Quality of life change, mean (SD)**	−2.2 (5.6)	−0.8 (5.2)

These tables present observed descriptive summaries. Inferential longitudinal comparisons are based on the treatment-by-time interaction from the mixed-effects models reported in the Results; therefore no timepoint-specific *P* value is used as the primary inference.

Abbreviations: ADC, antibody-drug conjugate; min, minutes; NRS, Numerical Rating Scale; SD, standard deviation.

a Percentages calculated from those completing 3-month assessment.

#### Anthropometric changes

ADC patients experienced greater observed weight loss (mean −3.1 kg, SD 1.4 vs −1.2 kg, SD 1.1). Clinically significant weight loss (≥5% baseline) occurred in 21/48 (43.8%) of ADC patients vs 4/70 (5.7%) of chemotherapy patients.

#### Lifestyle factors

Physical activity declined in both cohorts; 14.6% of ADC and 24.3% of chemotherapy patients met activity guidelines at T1 (Fisher’s exact *P* = .248). The mean change in weekly moderate-intensity activity was −41 minutes in the ADC group vs −22 minutes in the chemotherapy group. Poor sleep quality was reported by 41.7% of ADC and 28.6% of chemotherapy patients at T1 (Fisher’s exact *P* = .168).

#### Symptom burden

Pain intensity at 3 months was lower in the ADC cohort than in the chemotherapy cohort (mean NRS 3.7, SD 1.9 vs 5.3, SD 2.1), reflecting a smaller mean increase from baseline in the ADC group (+0.2) compared to the chemotherapy group (+1.4). This timepoint contrast is descriptive; the corresponding longitudinal inference is the treatment-by-time interaction reported below.

#### Psychosocial wellbeing

Patients receiving ADCs had higher observed stress levels at 3 months (mean 14.1, SD 4.6 vs 11.5, SD 4.0). Anxiety scores were 9.1 (SD 4.3) vs 7.2 (SD 4.1); these observed timepoint differences were descriptive and were not used as the primary longitudinal inference. Social relationship quality did not show a statistically significant between-cohort difference at T1.

### Caregiver burden

Temporal assistance burden increased more for ADC caregivers (mean change +3.4, SD 6.9 vs +1.3, SD 5.5; *P* = .08), without conversion to an estimated number of weekly caregiving hours. Physical fatigue increased more with ADC caregiving (mean change +2.4, SD 5.2 vs +0.8, SD 5.0; *P* = .10). Caregiver quality of life declined more in the ADC cohort (mean change −2.2, SD 5.6 vs −0.8, SD 5.2; *P* = .17). These between-cohort differences in observed change scores did not reach statistical significance; the corresponding longitudinal inference is given by the mixed-effects treatment-by-time interaction reported below.

#### Sensitivity analysis excluding T-DM1

Repeating the longitudinal model after excluding the 19 T-DM1-treated patients (remaining ADC cohort: T-DXd *n* = 9 and sacituzumab govitecan *n* = 23; 291 PROM observations; Table S3) yielded treatment-by-time interactions at T1 of −1.31 (95% CI −2.58 to −0.03; *P* = .044) for pain, +1.71 (−0.36 to 3.79; *P* = .105) for stress, +0.85 (−0.95 to 2.64; *P* = .356) for anxiety, +0.16 (−1.71 to 2.04; *P* = .865) for caregiver temporal assistance, −2.64 (−4.65 to −0.63; *P* = .010) for caregiver quality of life, +2.77 (0.93 to 4.61; *P* = .003) for caregiver physical fatigue, −1.06 (−2.98 to 0.86; *P* = .278) for caregiver social functioning, −1.50 (−3.43 to 0.43; *P* = .127) for caregiver emotional wellbeing, and −0.46 (−1.27 to 0.35; *P* = .267) for total caregiver burden. At T2, the corresponding estimates were −0.60 (−1.92 to 0.73; *P* = .377), +1.38 (−0.77 to 3.53; *P* = .208), −0.46 (−2.32 to 1.41; *P* = .630), −0.70 (−2.66 to 1.25; *P* = .479), −1.57 (−3.65 to 0.52; *P* = .140), +2.43 (0.52-4.33; *P* = .013), −0.42 (−2.41 to 1.57; *P* = .677), −0.76 (−2.76 to 1.24; *P* = .456), and −0.22 (−1.07 to 0.62; *P* = .606), respectively. The pattern was not uniformly reversed, but estimates were less precise.

### Six-month outcomes: longitudinal trajectories

By 6 months, several metrics showed attenuation from their 3-month values, although the longitudinal pattern was heterogeneous across domains (Table 4).

**Table 4.** Six-month outcomes: observed descriptive summaries.

Outcome	ADC (*n* = 44)	Standard Chemo (*n* = 64)
**Anthropometric**		
** Weight change from baseline, mean (SD), kg**	−2.1 (1.6)	−0.8 (1.2)
**Physical activity**		
** Meeting guidelines (≥150 min/wk), *n* (%)**	7 (15.9)	22 (34.4)
**Symptom burden**		
** Pain NRS, mean (SD)**	3.5 (1.6)	4.4 (1.9)
** Δ from 3-month assessment**	−0.2	−0.9
**Psychosocial**		
** Stress score, mean (SD)**	12.8 (4.4)	10.9 (3.8)
** Δ from 3-month assessment**	−1.3	−0.6
** Anxiety score, mean (SD)**	7.8 (3.9)	6.9 (3.7)
** Δ from 3-month assessment**	−1.3	−0.3
**Caregiver burden**		
** Temporal assistance, mean (SD)**	11.2 (4.3)	9.1 (3.7)
** Physical fatigue, mean (SD)**	9.0 (3.0)	7.4 (3.0)
** Quality of life, mean (SD)**	11.5 (3.4)	13.2 (3.4)
** Social functioning, mean (SD)**	11.0 (3.2)	12.4 (3.2)
** Emotional wellbeing, mean (SD)**	10.2 (3.2)	11.7 (3.2)

These tables present observed descriptive summaries. Inferential longitudinal comparisons are based on the treatment-by-time interaction from the mixed-effects models reported in the Results; therefore, no timepoint-specific *P* value is used as the primary inference.

Abbreviations: ADC, antibody–drug conjugate; NRS, Numerical Rating Scale; SD, standard deviation; Δ, change.

#### Anthropometric stabilization

Weight loss partially stabilized in the ADC cohort (mean −2.1 kg from baseline at 6 months vs −3.1 kg at 3 months) and remained greater than in the chemotherapy cohort (−0.8 kg). These timepoint summaries are descriptive; formal longitudinal inference is based on the treatment-by-time interaction.

#### Lifestyle

Physical activity remained low in the ADC cohort at 6 months: 15.9% of ADC patients met guidelines at 6 months (vs 14.6% at 3 months), compared with 34.4% in the chemotherapy cohort (Fisher’s exact *P* = .046). Poor sleep quality improved in both cohorts, reported by 20.5% of ADC and 21.9% of chemotherapy patients at 6 months (Fisher’s exact *P* = 1.00).

#### Symptom moderation

Pain intensity slowly decreased from 3-month peak in ADC cohort (mean NRS 3.5, SD 1.6 at 6 months vs 3.7 at 3 months); the longitudinal interaction did not show a significant T2 difference.

#### Psychosocial improvement

Stress levels decreased from the T1 value in ADC patients (mean 12.8, SD 4.4 vs 14.1 at T1); the mixed-effects model did not show a statistically significant T2 treatment-by-time interaction. Anxiety showed no significant treatment-by-time interaction at either follow-up timepoint.

#### Caregiver burden stabilization

Temporal assistance burden was 11.2 (SD 4.3) at T2 in the ADC cohort vs 9.1 (SD 3.7) in the chemotherapy cohort; the mixed-effects model did not show a significant T2 treatment-by-time interaction. Total caregiver burden likewise showed no significant treatment-by-time interaction.

#### Longitudinal mixed-effects analysis

In the primary models, the ADC-vs-chemotherapy treatment-by-time interaction at T1 was −1.09 for pain (95% CI, −2.18 to −0.01; *P* = .048), +2.11 for stress (95% CI, 0.32-3.91; *P* = .021), +2.51 for caregiver physical fatigue (95% CI, 0.97-4.06; *P* = .001), and −1.69 for caregiver quality of life (95% CI, −3.41 to 0.02; *P* = .053); other T1 interactions were not statistically significant. At T2, caregiver physical fatigue remained higher in the ADC cohort by the interaction estimate (+1.92; 95% CI, 0.33-3.50; *P* = .018), whereas other T2 interactions were not statistically significant. These estimates describe longitudinal associations and do not establish causal effects.

### Correlations: lifestyle factors and psychosocial outcomes

In pooled descriptive analyses (348 observations; Table 5), weekly physical activity showed little association with pain (Spearman *r* = 0.03, *P* = .56), anxiety (*r* = −0.02, *P* = .73), caregiver emotional wellbeing (*r* = 0.00, *P* = .97), or sleep duration (*r* = 0.04, *P* = .47). A small inverse association with stress was observed (*r* = −0.12, *P* = .03). These exploratory pooled correlations should not be interpreted causally. In the per-timepoint estimates (Table S1), the only association reaching nominal significance was a positive correlation between weekly physical activity and pain at 3 months (*r* = +0.24, *P* = .01), which is not evident in the pooled estimate; the discrepancy reflects pooling across timepoints and is interpreted as exploratory.

**Table 5.** Correlations between physical activity and psychosocial outcomes (all patients, all timepoints).

Outcome variable	Correlation with weekly physical activity (min)	*P*-value
**Pain NRS**	*r* = 0.03	.56
**Stress score**	*r* = −0.12	.03
**Anxiety score**	*r* = −0.02	.73
**Caregiver emotional wellbeing**	*r* = 0.00	.97
**Sleep duration (hr)**	*r* = 0.04	.47

Spearman correlation coefficients; *n* = 348 observations (baseline + follow-up assessments) Abbreviation: NRS, Numerical Rating Scale.

### Treatment discontinuation

By 6 months, 14 patients (7 in each cohort) had discontinued treatment, most commonly because of disease progression (9 of 14 patients; Table 6).

**Table 6.** Reasons for treatment discontinuation by 6 months.

Reason	ADC (*n* = 7)	Standard Chemo (*n* = 7)	Total (*n* = 14)
**Disease progression**	5 (71.4%)	4 (57.1%)	9 (64.3%)
**Toxicity/intolerance**	1 (14.3%)	2 (28.6%)	3 (21.4%)
**Death**	1 (14.3%)	1 (14.3%)	2 (14.3%)

Treatment discontinuation did not automatically imply loss from PROM follow-up; subsequent PROM assessment was obtained when clinically feasible. Missing PROM assessments and documented intercurrent events are summarized separately in Table S2.

## Discussion

This prospective real-world study provides a comprehensive multidimensional description of patients with MBC receiving ADC vs conventional chemotherapy using systematic ePROM integration. Three principal findings emerge with important clinical implications.

Patients in the ADC cohort showed greater observed weight loss, a steeper decline in physical activity, and higher values in selected psychosocial and caregiver domains at 3 months; these between-cohort findings are exploratory and cannot be interpreted as causal effects of ADC treatment. The observed differences may reflect treatment-related toxicity, tumor biology, clinical selection, supportive care, or other unmeasured factors; these mechanisms were not directly tested in this study. At T1, 21/48 (43.8%) ADC patients vs 4/70 (5.7%) chemotherapy patients had ≥5% weight loss.

The substantial physical activity decline (from 43.1% at baseline to 14.6% at T1 in the ADC cohort) has concerning implications, given evidence that maintained exercise during cancer treatment improves quality of life and reduces fatigue.^26^^,^^27^ The decline observed in patients receiving ADCs may reflect treatment toxicity, disease burden, treatment selection, or other factors; the present observational design cannot establish a treatment-specific mechanism.

Caregiver temporal assistance and physical fatigue increased from baseline to 3 months in both cohorts, with larger descriptive changes in the ADC group. These findings should be interpreted as exploratory longitudinal observations rather than causal treatment effects.

Despite the early burden observed in the ADC cohort, several metrics attenuated from their 3-month values by 6 months. This pattern was heterogeneous and should not be interpreted as evidence of physiological or psychological adaptation because attrition, disease course, treatment exposure, supportive care, and other unmeasured factors may contribute.

Our real-world findings align with patient-reported outcome (PRO) data from pivotal ADC randomized trials, which collectively show preservation of global quality of life (QoL) despite treatment-specific symptom burdens.^28–34^ Across the DESTINY-Breast program, trastuzumab deruxtecan (T-DXd) maintained EORTC QLQ-C30 global health/QoL scores and delayed time to definitive deterioration vs physician’s choice chemotherapy or T-DM1, with clinically meaningful improvements in pain, fatigue and physical, role and emotional functioning, even with longer treatment duration.^28–30^ Gastrointestinal toxicities (nausea, vomiting, appetite loss, constipation) remained the principal domains of QoL deterioration in DESTINY-Breast04 and -Breast06, underscoring the need for proactive antiemetic prophylaxis.^29^^,^^30^ T-DM1 data from EMILIA and KATHERINE similarly showed maintenance of global QoL with transient worsening in fatigue, GI symptoms and role functioning that attenuated over time, consistent with attenuation of some observed differences over time in our cohort,^33^^,^^34^ although formal adaptation was not tested.

For sacituzumab govitecan (SG), TROPiCS-02 and ASCENT PRO analyses demonstrated broad health-related QoL benefit vs physician’s choice, with significantly longer time to first clinically meaningful worsening across global health, physical and emotional functioning, fatigue, dyspnea, insomnia, and financial difficulties.^31^^,^^32^ Diarrhea emerged as the consistent limiting toxicity in both PRO-CTCAE and EORTC domains, mirroring the gastrointestinal burden observed in our real-world ADC cohort.^31^^,^^32^ Our real-world data extend this trial-derived evidence by demonstrating that, outside controlled trial populations, the early months of ADC therapy carry a pronounced multidimensional burden (weight loss, physical inactivity, psychological distress, and caregiver strain) rarely captured in pivotal trials. Some measures showed attenuation at 6 months, but the longitudinal models did not establish a uniform pattern of recovery or adaptation. These findings support monitoring early patient experience without implying a treatment-specific mechanism.

It is important to acknowledge that the observed differences between cohorts reflect not only the distinct pharmacological profiles of ADCs vs standard chemotherapy but also the inherently different patient populations being compared. Patients receiving ADCs were exclusively those with HER2-positive or triple-negative disease, subtypes associated with more aggressive clinical behaviors and a higher symptom burden. In contrast, 45% of patients receiving standard chemotherapy had hormone receptor-positive/HER2-negative disease, a subtype typically characterized by a more indolent course. These biological differences may independently influence quality-of-life trajectories, lifestyle patterns, and caregiver burden irrespective of the treatment received. Accordingly, the between-group differences described herein should be interpreted as reflecting clinically distinct patient populations, rather than as direct causal effects of one treatment modality over another.

Several mechanisms may contribute to the observed 3-to-6-month attenuation, including changes in treatment exposure, supportive care, disease burden, and selective continuation; however, these mechanisms were not formally tested in the present study and causal attribution is not possible.

Strengths include the prospective design, consecutive enrollment, comprehensive multidimensional assessment, high follow-up completion, pragmatic real-world setting and concurrent assessment of 122 primary caregivers, enabling dyadic patient–caregiver analysis. The DAMA framework also demonstrates the feasibility of systematic ePROM integration in a nonacademic community Breast Unit. Limitations should be acknowledged. The observational design and treatment assignment based on tumor biology preclude causal inference, and formal multivariable adjustment was not undertaken. The ADC and chemotherapy cohorts inevitably encompassed heterogeneous tumor subtypes (HER2-positive, HR+/HER2-negative, TNBC) with distinct prognoses and intrinsic QoL trajectories; although biology-driven assignment was unavoidable, residual confounding by subtype cannot be excluded and should be addressed in larger, subtype-stratified cohorts. The single-center Italian setting may limit generalizability. The use of mixed-effects longitudinal models represents the primary repeated-measures analysis; remaining analytical limitations include the exploratory nature of the between-cohort comparison and the descriptive correlation analyses. The 6-month endpoint may not capture longer-term trajectories; non-completion of baseline ePROMs by 18.7% of patients (more frequent at worse performance status and lower education) suggests selection bias toward healthier patients; and financial toxicity and disease-specific symptom subtypes beyond pain were not systematically captured. Most importantly, this study describes associations between ePROMs and outcomes but does not test whether systematic ePROM collection with triggered interventions improves outcomes. Randomized trials of DAMA-driven supportive interventions vs usual care, predictive models incorporating baseline PROMs, implementation-science studies, and health-economic analyses are needed to establish clinical and cost-effectiveness benefit and to define optimal workflows for routine MBC care.

In this prospective real-world cohort from a community-based Italian breast unit, ADC and standard intravenous chemotherapy cohorts differed in several patient- and caregiver-reported outcomes during the first 6 months, with greater observed weight loss and physical-activity decline and higher caregiver burden in the ADC cohort, but lower observed pain at 3 months. Because treatment assignment was biology- and clinically driven, these findings should not be interpreted as causal effects of ADC treatment. Several measures attenuated by 6 months, but the longitudinal models did not establish a uniform pattern of recovery or adaptation. Pooled exploratory correlations between physical activity and patient/caregiver outcomes were generally weak, with only a small inverse association with stress. Systematic patient-reported outcome measure implementation via the DAMA framework enabled comprehensive burden characterization and identification of patients requiring supportive care, demonstrating feasibility in routine practice.

## Supplementary Material

oyag394_Supplementary_Data

## Data Availability

The datasets generated and/or analyzed during the current study are not publicly available due to ethical restrictions imposed by the local Ethics Committee (Comitato Etico Territoriale, ASST Cremona) and patient privacy protection requirements under the EU General Data Protection Regulation (GDPR, Reg. EU 2016/679) but are available from the corresponding author on reasonable request.
