# Supplementary material for: Real-world patient-reported outcome comparing antibody–drug conjugates and conventional chemotherapy in metastatic breast cancer
Source: Oncologist. 2026 Oct 5;31(11):oyag394. doi: 10.1093/oncolo/oyag394 (PMC13694110; doi:10.1093/oncolo/oyag394)
Supplement: Supplementary file 1 [file oyag394_supplementary_data.zip › DAMA_Supplemental_Table_S1_160926.docx]

**Supplemental Table S1. Per-timepoint Spearman correlations between weekly moderate-intensity physical activity and patient and caregiver outcomes**

Values are Spearman rank correlation coefficients with two-tailed p values in parentheses. Coefficients were estimated separately at each timepoint to avoid pseudo-replication; the pooled estimates across all 348 observations are reported in Table 5 of the main manuscript. No global multiplicity correction was applied because these analyses were exploratory.

| **Outcome variable** | **T0 (n=122)** | **T1 (n=118)** | **T2 (n=108)** |
| --- | --- | --- | --- |
| Pain NRS | +0.05 (0.56) | +0.24 (0.01) | −0.07 (0.46) |
| Stress score | −0.04 (0.68) | −0.15 (0.11) | −0.16 (0.09) |
| Anxiety (GAD-7 total score) | +0.08 (0.39) | +0.01 (0.94) | +0.03 (0.78) |
| Caregiver emotional wellbeing | −0.12 (0.18) | +0.08 (0.41) | +0.10 (0.32) |
| Sleep duration | −0.06 (0.51) | +0.05 (0.59) | −0.06 (0.55) |

*Abbreviations: NRS, Numerical Rating Scale; GAD-7, Generalized Anxiety Disorder-7; T0, baseline; T1, 3-month assessment; T2, 6-month assessment.*
