# Supplementary material for: Real-world patient-reported outcome comparing antibody–drug conjugates and conventional chemotherapy in metastatic breast cancer
Source: Oncologist. 2026 Oct 5;31(11):oyag394. doi: 10.1093/oncolo/oyag394 (PMC13694110; doi:10.1093/oncolo/oyag394)
Supplement: Supplementary file 1 [file oyag394_supplementary_data.zip › DAMA_Supplemental_Table_S2_160926.docx]

**Supplemental Table S2. Missing PROM assessments and intercurrent events**

Observed PROM denominators and documented treatment-discontinuation/intercurrent events through 6 months. Missing PROM assessment and treatment discontinuation are treated as distinct constructs.

| Timepoint | Treatment group | Expected PROM N | Observed PROM N | Missing PROM N (cumulative) | Discontinuation: progression | Discontinuation: death | Discontinuation: toxicity/intolerance |
| --- | --- | --- | --- | --- | --- | --- | --- |
| T0 | ADC | 51 | 51 | 0 | 0 | 0 | 0 |
| T0 | Chemotherapy | 71 | 71 | 0 | 0 | 0 | 0 |
| T1 | ADC | 51 | 48 | 3 | 3 | 0 | 0 |
| T1 | Chemotherapy | 71 | 70 | 1 | 1 | 0 | 0 |
| T2 | ADC | 51 | 44 | 7 | 2 | 1 | 1 |
| T2 | Chemotherapy | 71 | 64 | 7 | 3 | 1 | 2 |

Notes. The expected PROM denominator is the baseline analysis cohort within each treatment group. The observed denominators are 122 at T0, 118 at T1 and 108 at T2. The 14 patients without a later PROM assessment were classified in the manuscript as disease progression (n=7) or loss to follow-up (n=7), comprising 3 progression and 4 loss to follow-up in ADC patients and 4 progression and 3 loss to follow-up in chemotherapy patients; those follow-up non-completion categories are not assumed to correspond one-to-one with the treatment-discontinuation events shown above. The intercurrent-event counts are based on documented treatment-discontinuation records in the analysis dataset. Treatment discontinuation did not automatically imply loss from PROM follow-up. Discontinuation events are counted in the interval preceding each timepoint and therefore sum across rows to the totals reported in Table 6 (ADC: 5 progression, 1 toxicity/intolerance, 1 death; chemotherapy: 4 progression, 2 toxicity/intolerance, 1 death). Missing PROM N is cumulative relative to the baseline analysis cohort.

Clarification: Missing PROM assessments at T2 were classified as follows: ADC, 7 missing (3 due to disease progression and 4 due to loss to follow-up); conventional chemotherapy, 7 missing (4 due to disease progression and 3 due to loss to follow-up). These PROM non-completion categories are distinct from treatment-discontinuation events reported in Table 6.
