# Supplementary material for: Real-world patient-reported outcome comparing antibody–drug conjugates and conventional chemotherapy in metastatic breast cancer
Source: Oncologist. 2026 Oct 5;31(11):oyag394. doi: 10.1093/oncolo/oyag394 (PMC13694110; doi:10.1093/oncolo/oyag394)
Supplement: Supplementary file 1 [file oyag394_supplementary_data.zip › DAMA_Supplemental_Table_S3_160926.docx]

**Supplemental Table S3. Sensitivity analysis excluding T-DM1**

Treatment-by-time interaction estimates from linear mixed-effects models after exclusion of the 19 patients treated with T-DM1. The sensitivity cohort comprised 32 ADC-treated patients (trastuzumab deruxtecan, n=9; sacituzumab govitecan, n=23), contributing 291 PROM observations. Estimates are for ADC (T-DXd + sacituzumab govitecan) versus conventional chemotherapy; T0 is the reference timepoint. Models included a patient-level random intercept, categorical time, treatment group, and the time-by-treatment interaction. Negative estimates indicate a lower longitudinal value in the ADC cohort relative to chemotherapy at the specified timepoint interaction.

| Outcome | T1 interaction estimate (95% CI) | P | T2 interaction estimate (95% CI) | P |
| --- | --- | --- | --- | --- |
| Pain (0–10) | −1.308 (−2.582 to −0.033) | 0.044 | −0.595 (−1.915 to 0.726) | 0.377 |
| Stress (1–20) | +1.714 (−0.358 to 3.786) | 0.105 | +1.379 (−0.768 to 3.526) | 0.208 |
| Anxiety (GAD-7, 0–21) | +0.846 (−0.951 to 2.642) | 0.356 | −0.459 (−2.322 to 1.405) | 0.630 |
| Caregiver temporal assistance | +0.162 (−1.711 to 2.036) | 0.865 | −0.705 (−2.657 to 1.248) | 0.479 |
| Caregiver quality of life | −2.636 (−4.646 to −0.626) | 0.010 | −1.568 (−3.652 to 0.515) | 0.140 |
| Caregiver physical fatigue | +2.766 (0.926 to 4.606) | 0.003 | +2.425 (0.517 to 4.334) | 0.013 |
| Caregiver social functioning | −1.060 (−2.976 to 0.857) | 0.278 | −0.423 (−2.411 to 1.565) | 0.677 |
| Caregiver emotional wellbeing | −1.502 (−3.432 to 0.428) | 0.127 | −0.761 (−2.762 to 1.240) | 0.456 |
| Total caregiver burden | −0.459 (−1.268 to 0.351) | 0.267 | −0.222 (−1.065 to 0.621) | 0.606 |

Values are estimates for the time-by-treatment interaction; 95% CIs are Wald confidence intervals. P values are nominal. The smaller sensitivity cohort reduces precision; results are interpreted as exploratory. The analysis did not adjust for tumor subtype because subtype was collinear with treatment assignment.
